# Growth in Total Height and Its Components and Cardiometabolic Health in Childhood

**DOI:** 10.1371/journal.pone.0163564

**Published:** 2016-09-22

**Authors:** Line Klingen Haugaard, Jennifer L. Baker, Wei Perng, Mandy Brown Belfort, Sheryl L. Rifas-Shiman, Karen Switkowski, Emily Oken, Matthew W. Gillman

**Affiliations:** 1 The Novo Nordisk Foundation Center for Basic Metabolic Research, Faculty of Health and Medical Sciences, University of Copenhagen, Copenhagen, Denmark; 2 Institute of Preventive Medicine, Bispebjerg and Frederiksberg Hospital, The Capital Region, Copenhagen, Denmark; 3 Department of Nutritional Sciences, University of Michigan, School of Public Health, Ann Arbor, Michigan, United States of America; 4 Department of Pediatric Newborn Medicine, Brigham and Women’s Hospital, Boston, Massachusetts, United States of America; 5 Obesity Prevention Program, Department of Population Medicine, Harvard Medical School and Harvard Pilgrim Health Care Institute, Boston, Massachusetts, United States of America; 6 Department of Nutrition, Harvard TH Chan School of Public Health, Boston, Massachusetts, United States of America; Mayo Clinic Arizona, UNITED STATES

## Abstract

**Background:**

Short stature or short legs is associated with cardiometabolic disease. Few studies have addressed this issue in children, incorporated repeated measures, or studied modern cohorts.

**Methods:**

We examined if change in total height, leg length and trunk length between two time points from early (median: 3.2 years) to mid-childhood (median: 7.7 years), with and without adjustment for concurrent change in adiposity (subscapular plus triceps skinfold thickness), was associated with mid-childhood cardiometabolic risk in 315 boys and 295 girls from Project Viva. The main outcome was a cardiometabolic risk score based on sex-specific internal z-scores for systolic blood pressure, waist circumference, homeostatic model assessment of insulin resistance, triglycerides and high-density lipoprotein-cholesterol.

**Results:**

Mean (SD) total height was 97.9 (4.5) cm in boys and 97.1 (4.7) cm in girls in early childhood and 129.1 (7.2) cm in boys and 128.3 (7.9) cm in girls in mid-childhood. Trunk length constituted about half of total height. In linear regression models adjusted for parental anthropometry and socio-demographics, faster growth in total height, leg length and particularly trunk length, were associated with higher cardiometabolic risk in mid-childhood. Per 1 cm annual increase in trunk length, the cardiometabolic risk score was 0.23 z-score (95% confidence interval [CI] 0.08, 0.39) higher among boys and 0.47 z-score (95% CI 0.33, 0.60) higher among girls. Estimates were attenuated after adjusting for adiposity (boys: 0.03 z-score, 95% CI -0.11, 0.18; girls: 0.32 z-score, 95% CI 0.19, 0.45).

**Conclusion:**

Rapid linear growth, particularly in trunk length, was associated with higher cardiometabolic risk in childhood, which was explained by relationships of linear growth with adiposity in boys, but only partly in girls.

## Introduction

Slower fetal and infant growth followed by faster growth during childhood is positively associated with a higher risk of later coronary heart disease (CHD) and diabetes [1–3]. Studies investigating growth have mainly focused on weight gain, whereas linear growth has received far less attention. A Finnish cohort study found positive associations of growth in total height from birth to age 7 years with risk of adult CHD in girls, but not in boys [4, 5]. Likewise, a Danish cohort study reported associations of growth in total height from 7 to13 years, in both sexes, with higher risk of adult CHD [6]. Similarly, investigators from the Avon Longitudinal Study of Parents and Children found positive relations between faster linear growth from infancy to age 10 years with systolic blood pressure at 10 years. However, studies in adults [7–14] and a few in children [15–17] have consistently shown that shorter leg length is associated with higher cardiometabolic risk. Because pre-pubertal linear growth occurs more in the legs than in the trunk, leg length may be a particularly sensitive indicator of early growth-influencing exposures such as nutrition, illness, and living circumstances [3, 18]. Therefore, assessing relations in childhood of height components with biomarkers of cardiometabolic risk, such as systolic blood pressure, waist circumference, insulin resistance and blood lipids may provide important insights into the origins of cardiometabolic disease.

Although all studies investigating height components aim to examine how pre-pubertal growth is associated with cardiometabolic risk, studies using repeated measures during childhood are lacking. Moreover, many of the prior studies included populations at substantial nutritional or socioeconomic risk, which may not be generalizable to modern populations in developed settings. Given the contradictory findings of both faster overall linear growth and shorter leg length being associated with higher cardiometabolic risk, we hypothesized that faster pre-pubertal growth, compared to normal growth, would be associated with higher cardiometabolic risk in childhood, and that these associations might be stronger for growth in trunk length than for growth in leg length. Furthermore, we hypothesized that the associations could be influenced by adiposity. We therefore investigated how growth in total height and its components between two time points in childhood were related to mid-childhood cardiometabolic risk, including whether the associations differed by concurrent changes in adiposity, in the Boston-area Project Viva cohort, a well-nourished population.

## Methods

Participants were enrolled in Project Viva, during early pregnancy between 1999 and 2002, from obstetric practices of Atrius Harvard Vanguard Medical Associates in eastern Massachusetts [19]. Of the 2128 singleton births, 992 attended an in-person visit in both early (median: 3.2 years) and mid-childhood (median: 7.7 years). From this analysis, we excluded children with mothers with a pre-pregnancy history of type I or type II diabetes (n = 6), whose gestational age at birth was less than 34 weeks (n = 13), and who reported diabetes (n = 2). Of these, we excluded 361 children who did not provide a blood sample at the mid-childhood visit. Thus our sample for analysis was 610 children (315 boys and 295 girls). Compared with the 610 participants in this analysis, the 1518 non-participants were somewhat less likely to have college-educated mothers (63% v. 68%) and to have annual household income exceeding $70,000 (57% v. 62%). However race/ethnicity, parental anthropometrics, and maternal age and marital status were similar. The study was approved by the Institutional Review Board of Harvard Pilgrim Health Care, and all procedures were conducted in accordance with established ethical standards. All mothers provided written informed consent, and children provided verbal assent at mid-childhood.

### Anthropometric measurements

At in-person visits, trained research assistants measured standing and sitting heights to the nearest 0.1 cm using a wooden stadiometer (Shorr Productions, Olney, MD). We calculated leg length as the difference between standing and sitting heights. The research assistants also measured weight to the nearest 0.1 kg using an electronic scale (early childhood visit: Seca model 881, Seca Corporation, Hanover, Maryland, USA; mid-childhood visit: Tanita, Arlington Heights, IL), waist circumference at the level of the iliac crest using a non-stretchable measuring tape (Hoechstmass Balzer GmbH, Sulzbach, Germany), and subscapular and triceps skinfold thickness with Holtain calipers (Crosswell, Crymych, Pembrokeshire, UK). We calculated the sum of the two skinfold thicknesses to estimate overall adiposity.

### Cardiometabolic risk parameters

For each child, we obtained up to five blood pressure measurements at one-minute intervals using an automated oscillometric recorder (Dinamap Critikon, Tampa, FL). The collection and storage of blood samples have been reported elsewhere [19, 20]. We measured fasting insulin using an electrochemiluminescence immunoassay. Fasting glucose was measured enzymatically (Roche Diagnostics, Indianapolis, IN). We calculated insulin resistance by using the homeostatic model assessment (HOMA-IR = fasting insulin [μU/mL] x fasting glucose [mg/dL]/405). Triglycerides and HDL-cholesterol were measured enzymatically with correction for endogenous glycerol.

The main outcome was a mid-childhood cardiometabolic risk score [20–22] derived as the mean of five sex-specific z-scores for systolic blood pressure, waist circumference, log-transformed HOMA-IR, log-transformed triglycerides and HDL-cholesterol (scaled inversely). A positive score indicates a higher cardiometabolic risk and a negative score indicates a lower cardiometabolic risk than the cohort average. We also examined components included in the cardiometabolic risk score individually.

### Additional participant characteristics

At enrollment, we collected information on maternal age, height, pre-pregnancy weight, date of last menstrual period, education, marital status, household income and child’s sex via questionnaires and interviews. Mothers also provided information on the father’s height and weight. We calculated body mass index (BMI, kg/m^2^) for both parents. From hospital delivery records we collected information on sex, birthweight and delivery date. At the early childhood visit, parents reported the child’s race/ethnicity by choosing one or more of the following racial/ethnic groups when asked: “Which of the following best describes your child’s race or ethnicity?”: Hispanic or Latina, white or Caucasian, black or African American, Asian or Pacific Islander, American Indian or Alaskan Native, and other (please specify). At the mid-childhood visit, mothers reported signs of puberty, and we determined pubertal status on the basis of whether the mothers answered “no”, “barely started”, or “yes, definitely/complete” to development of body hair or breasts in girls, and in boys to development of body hair, deepening of voice, or development of facial hair.

### Statistical analysis

We examined the correlations between early and mid-childhood anthropometrics using Spearman correlations. Given potential sex differences in cardiometabolic risk and childhood growth, we conducted analyses separately for boys and girls [23, 24], even though we only found sex-interactions for the associations between growth in leg length and cardiometabolic risk score (*p* = 0.02 when using the likelihood ratio test). We first examined bivariate relationships of early to mid-childhood growth in total height, leg length and trunk length, each expressed as cm/year, as well as early to mid-childhood change in adiposity (change in subscapular plus triceps skinfold thickness [mm/year]), with the mid-childhood cardiometabolic risk score. We then used multivariable linear regression models to examine associations of growth in total height and its components with cardiometabolic risk score and the individual cardiometabolic biomarkers. Before entering exposures as continuous variables in regression models, we ensured linearity of associations by examining exposures in sex-specific quartiles.

In the multivariable linear regression analysis, we started with base models including child’s age at each visit, child’s race/ethnicity, and baseline (early childhood) total height, leg or trunk length (Model 1). In the models for annual growth in leg length and trunk length we then added growth in the other height component to investigate the independent contributions of each (Model 2). Additionally, we adjusted for height and BMI of both parents (Model 3), and our final model also included marital status in pregnancy and level of maternal education, as potential socioeconomic confounding factors (Model 4). Higher effect estimates for the association between growth in total height and mid-childhood cardiometabolic risk score were observed for both sexes when excluding baseline total height in the model (S1 Table). This could potentially be caused by multicollinearity in the model between baseline total height and growth in total height. However, the widths of the confidence intervals were similar, which is not expected in the presence of multicollinearity. The change in effect estimate seen with the inclusion of baseline total height in our model could then be because baseline height also influences cardiometabolic risk. As the exclusion of baseline total height did not change our overall conclusions, and because dropping baseline leg or trunk length only minimally affected their associations with the cardiometabolic risk score, we included the respective baseline height component in our models. The interpretation is thus that we, in our models, investigate the effect of linear growth on cardiometabolic risk regardless of the child’s starting point. We also considered adjustment for birth weight for gestational age, household income, maternal pregnancy smoking status, duration of breastfeeding as well as pubertal status but their inclusion in the final model did not change estimated associations or conclusions. Thus we did not include them in the analyses.

As in our previous work investigating height components in Project Viva, we based our conclusions on models that included growth in ‘absolute’ leg length and trunk length [17]. Some studies have used ratios (e.g. sitting height ratio and leg-to-trunk ratio), which are relevant when used in a clinical setting, but it can be challenging to interpret their coefficients when they are used in a regression, as e.g. a positive association of leg-to-trunk ratio with a chosen outcome could be due to either an increase in leg length or a decrease in trunk length.

As children with faster linear growth may accumulate more adipose tissue (S2 Table), we assessed the extent to which change in overall adiposity explained our findings by adding annual change in sum of skinfold thicknesses in the final model. We included all the cardiometabolic outcomes in native, untransformed, units after having examined the residuals from each model to make sure that the assumption of all cardiometabolic outcomes being normally distributed was reasonable.

We performed a series of sensitivity analyses: We investigated associations of total height and its components with systolic blood pressure and waist circumference in the larger group of children with at least one available outcome (n = 971), and as we found similar results we report everything for the smaller subgroup with additional information on blood outcomes (n = 610). As black children typically have longer legs than white children of the same age [3], we conducted an analysis for black (59 boys and 51 girls) and white (196 boys and 189 girls) children separately. The estimates were generally similar and we chose to adjust rather than stratify by race in our main analysis. Due to very low numbers of other race/ethnicities these were not included in the analysis.

To account for missing data, we performed multiple imputation for all 2128 mother-child pairs in Project Viva. We then limited the analysis to the 610 included participants. We provided information about the number of imputed values for all variables along with descriptive data for the 470 participants in the complete case analysis in S3 Table. We used SAS (Proc MI) to impute 50 values for each missing observation and combined multivariable modeling estimates using Proc MI ANALYZE in SAS version 9.3 (SAS Institute, Cary NC). An alternative approach, using only participants with all covariate data (complete case), yielded similar results.

## Results

We included 315 boys and 295 girls in the main analyses (Table 1). Boys were on average 0.8 cm taller than girls at both the early and the mid-childhood visit. Between the two visits (∆time: 2.0–7.5 years for boys, 1.3–7.4 years for girls) the annual growth in leg length and trunk length were almost the same in boys and girls, and as expected, gain in leg length (4.0 cm/y in both sexes) was larger than gain in trunk length (2.7 cm/y in boys and 2.9 cm/y in girls). The correlation between leg and trunk length was stronger in mid-childhood than in early childhood (Spearman correlation coefficients 0.62 and 0.42, respectively). Girls had similar blood pressure, but a greater increase in sum of skinfold thicknesses, higher levels of HOMA-IR and triglycerides, and lower levels of HDL-cholesterol than boys, and in mid-childhood more girls than boys had evidence of having entered puberty (11.0% vs. 6.9%). Additional information on the anthropometric and age variables as well as their mutual correlation coefficients is provided in S4 and S5 Tables.

**Table 1:** Parental and child characteristics of 610 Project Viva participants.

	Boys (n = 315)	Girls (n = 295)
	% or Mean (SD)	
**Parental and family characteristics**		
Maternal age at enrollment, years	31.9 (5.6)	32.4 (5.3)
Maternal height, cm	165.4 (7.2)	165.1 (7.0)
Maternal pre-pregnancy BMI, kg/m^2^	24.6 (5.2)	25.1 (5.2)
Paternal height, cm	179.4 (7.5)	179.2 (7.9)
Paternal BMI, kg/m^2^	26.0 (3.7)	26.8 (3.8)
Maternal education		
< College grad	34.0%	29.8%
≥ College grad	66.0%	70.2%
Marital status		
Single	8.6%	10.6%
Married/cohabiting	91.4%	89.4%
Annual household income		
≤ $70,000	39.4%	36.9%
> $70,000	60.6%	63.1%
**Early childhood visit** (median age 3.2 years)		
Child’s race/ethnicity		
Black	18.7%	17.3%
Hispanic	5.1%	3.7%
White	62.2%	64.1%
Asian	2.5%	2.4%
Other	11.4%	12.5%
Total height, cm	97.9 (4.5)	97.1 (4.7)
Leg length, cm	42.1 (2.8)	41.9 (2.9)
Trunk length, cm	55.8 (2.6)	55.3 (2.5)
Subscapular + triceps skinfold thicknesses, mm	16.0 (3.9)	17.4 (4.4)
**Mid-childhood visit** (median age 7.7 years)		
Total height, cm	129.1 (7.2)	128.3 (7.9)
Leg length, cm	60.6 (4.7)	60.1 (5.1)
Trunk length, cm	68.5 (3.4)	68.2 (3.6)
Subscapular + triceps skinfold thickness, mm	17.6 (8.2)	21.8 (10.1)
Time between early and mid-childhood visit, years	4.6 (0.8)	4.5 (0.7)
Change in total height, cm/y	6.7 (0.7)	6.9 (0.7)
Change in leg length, cm/y	4.0 (0.6)	4.0 (0.5)
Change in trunk length, cm/y	2.7 (0.4)	2.9 (0.5)
Change in subscapular + triceps skinfold thickness, mm/y	0.3 (1.5)	0.9 (1.8)
Pubertal status in mid-childhood,		
No	81.4%	70.0%
Maybe (barely started)	11.8%	19.0%
Yes	6.9%	11.0%
Cardiometabolic outcomes		
Systolic blood pressure, mmHg	94.3 (8.7)	94.3 (8.7)
Waist circumference, cm	59.4 (7.9)	60.1 (8.5)
HOMA-IR, units	1.6 (1.5)	1.9 (1.5)
Triglycerides, mg/dL	57.4 (24.3)	58.8 (25.1)
HDL-cholesterol, mg/dL	58.5 (13.1)	55.7 (13.7)
Cardiometabolic risk score	0.00 (0.59)	0.00 (0.64)

Abbreviations: BMI, body mass index; HDL, high-density lipoprotein; HOMA-IR, homeostatic model assessment of insulin resistance; y, year. Change is calculated as the difference in the respective variable between early and mid-childhood visit, divided by the time elapsed in years. The cardiometabolic risk score is composed of the mean of five sex-specific internal z-scores for systolic blood pressure, waist circumference, log-transformed HOMA-IR, log-transformed triglycerides and inverted HDL-cholesterol.

By definition, the mean cohort-specific cardiometabolic risk score was 0. When investigating quartiles of linear growth we found that boys who were in the 3^rd^ quartile and girls who were in the 4^th^ quartile of growth in trunk length had a mean mid-childhood cardiometabolic risk score of 0.12 and 0.27 z-score, respectively, which were the highest scores observed across the quartiles of linear growth (Table 2). Children in the 4^th^ quartile of change in overall adiposity had the highest observed cardiometabolic risk scores (boys: 0.50 z-score; girls: 0.56 z-score).

**Table 2:** Mid-childhood cardiometabolic risk score according to quartiles of annual change in total height, its components, and adiposity from early to mid-childhood (610 participants from Project Viva).

	Boys (n = 315)			Girls (n = 295)		
	Range (cm/y or mm/y)		Cardiometabolic risk score	Range (cm/y or mm/y)		Cardiometabolic risk score
	Min	max	Mean (SD)	min	max	Mean (SD)
**Total height and its components**						
Growth in total height (cm/y)						
Quartile 1	2.21	6.31	-0.11(0.52)	5.14	6.36	-0.24 (0.61)
Quartile 2	6.32	6.74	-0.04 (0.50)	6.37	6.81	-0.10 (0.61)
Quartile 3	6.75	7.18	0.01 (0.60)	6.82	7.29	0.08 (0.66)
Quartile 4	7.18	8.73	0.14 (0.66)	7.30	9.46	0.26 (0.66)
Growth in leg length (cm/y)						
Quartile 1	0.20	3.63	-0.09 (0.51)	2.11	3.69	-0.12 (0.66)
Quartile 2	3.63	4.02	-0.02 (0.68)	3.70	3.99	-0.07 (0.59)
Quartile 3	4.02	4.37	0.05 (0.53)	4.00	4.30	0.02 (0.68)
Quartile 4	4.37	5.78	0.06 (0.59)	4.30	6.68	0.17 (0.60)
Growth in trunk length (cm/y)						
Quartile 1	0.99	2.50	-0.06 (0.51)	0.50	2.52	-0,23 (0.55)
Quartile 2	2.50	2.77	-0.08 (0.58)	2.52	2.82	-0.05 (0.57)
Quartile 3	2.77	2.99	0.12 (0.61)	2.83	3.17	0.01 (0.62)
Quartile 4	2.99	4.29	0.02 (0.61)	3.18	5.23	0.27 (0.71)
**Adiposity**						
Change in sum of subscapular and triceps skinfold thickness (mm/y)						
Quartile 1	-6.47	-0.49	-0.20 (0.47)	-2.72	-0.30	-0.32 (0.51)
Quartile 2	-0.49	0.00	-0.17 (0.43)	-0.29	0.41	-0.22 (0.44)
Quartile 3	0.03	0.68	-0.13 (0.40)	0.41	1.72	-0.01 (0.53)
Quartile 4	0.68	7.02	0.50 (0.68)	1.73	7.11	0.56 (0.67)

The cardiometabolic risk score is composed of the mean of five sex-specific internal z-scores for systolic blood pressure, waist circumference, log-transformed HOMA-IR, log-transformed triglycerides and inverted HDL-cholesterol.

In both sexes the minimally adjusted multivariable linear regression analyses showed that greater increases in total height, leg length, and trunk length were positively associated with mid-childhood cardiometabolic risk score (Table 3, Model 1). In Model 2, additional adjustment for growth in the other height component strengthened the association for growth in leg length in girls, whereas minor changes were observed in boys. The association for growth in trunk length remained the same in both sexes after adjustment for leg length. Further adjustment for parental anthropometrics slightly strengthened the associations for growth in leg length in girls, but did otherwise not affect the associations in both sexes (Model 3). Adjustment for socioeconomic factors had essentially no influence on the associations (Model 4).

**Table 3:** Multivariable linear regression models showing associations of growth in total height and its components from early to mid-childhood with cardiometabolic risk score in mid-childhood (610 participants from Project Viva).

	Change in cardiometabolic risk score (95% CI) per 1 cm annual growth							
	Model 1		Model 2		Model 3		Model 4	
	Β	95% CI	β	95% CI	β	95% CI	β	95% CI
**Boys (n = 315)**								
Growth in total height (cm/y)	0.10	-0.01, 0.20	-	-	0.09	-0.02, 0.20	0.10	-0.01, 0.21
Growth in leg length (cm/y)	0.15	0.04, 0.27	0.17	0.05, 0.28	0.15	0.03, 0.27	0.16	0.04, 0.28
Growth in trunk length (cm/y)	0.22	0.07, 0.38	0.23	0.07, 0.39	0.23	0.07, 0.39	0.23	0.08, 0.39
**Girls (n = 295)**								
Growth in total height (cm/y)	0.29	0.18, 0.40	-	-	0.29	0.18, 0.40	0.29	0.18, 0.40
Growth in leg length (cm/y)	0.18	0.04, 0.32	0.24	0.10, 0.37	0.22	0.09, 0.36	0.22	0.09, 0.36
Growth in trunk length (cm/y)	0.47	0.33, 0.61	0.47	0.33, 0.61	0.47	0.30, 0.60	0.47	0.33, 0.60

Abbreviations: CI, confidence interval. Model 1: Adjusted for child’s age at the early and mid-childhood visit, child’s race/ethnicity and baseline height component (early childhood). Model 2: Model 1 + rate of growth in other height component (i.e. rate of growth in leg length adjusted for rate of growth in trunk length and vice versa). Model 3: Model 2 + maternal pre-pregnancy body mass index and height and paternal body mass index and height. Model 4: Model 3 + maternal education and marital status in pregnancy. Growth is calculated as the difference in the respective variable between early and mid-childhood visit, divided by the time elapsed in years. The cardiometabolic risk score is composed of the mean of five sex-specific internal z-scores for systolic blood pressure, waist circumference, log-transformed HOMA-IR, log-transformed triglycerides and inverted HDL-cholesterol.

In general, associations with each of the five cardiometabolic outcomes were stronger for growth in trunk length than for growth in total height or leg length, although they were all largely in the same direction (Table 4). A 1 cm/year increase in trunk length among boys was associated with a 2.2 mmHg (95% confidence interval [CI] -0.2, 4.5) higher systolic blood pressure, a 4.8 cm (95% CI 3.0, 6.6) greater waist circumference, a 0.56 (95% CI 0.14, 0.97) higher HOMA-IR, a -0.57 mg/dL (95% CI -4.49, 3.36) lower HDL cholesterol-level, but a -2.06 mg/dL (95% CI -9.41, 5.29) lower level of triglycerides. In girls, a 1 cm/year increase in trunk length was associated with a 5.2 mmHg (95% CI 3.3, 7.1) higher systolic blood pressure, a 4.8 cm (95% CI 3.1, 6.4) greater waist circumference, a 0.86 (95% CI 0.50, 1.22) higher HOMA-IR, a 7.32 mg/dL (95% CI 0.95,13.70) higher level of triglycerides and a -4.31 mg/dL (95% CI -7.73, -0.90) lower HDL cholesterol-level.

**Table 4:** Associations of growth in total height and its components from early to mid-childhood with individual cardiometabolic outcomes in mid-childhood (610 participants from Project Viva).

	Estimates with 95% CI’s for cardiometabolic outcome per 1 cm annual growth									
	Systolic blood pressure		Waist Circumference		HOMA-IR		Triglycerides		HDL- cholesterol	
	(mmHg)		(cm)		(units)		(mg/dL)		(mg/dL)	
	Β	95% CI	β	95% CI	Β	95% CI	β	95% CI	β	95% CI
**Boys (n = 315)**										
Growth in total height (cm/y)	1.6	-0.1, 3.2	3.1	1.9, 4.3	0.23	-0.06, 0.52	-1.55	-6.71, 3.61	1.75	-0.92, 4.41
Growth in leg length (cm/y)	2.5	0.7, 4.4	4.1	2.7, 5.5	0.16	-0.17, 0.49	-0.45	-6.08, 5.19	1.88	-1.08, 4.83
Growth in trunk length (cm/y)	2.2	-0.2, 4.5	4.8	3.0, 6.6	0.56	0.14, 0.97	-2.06	-9.41, 5.29	-0.57	-4.49, 3.36
**Girls (n = 295)**										
Growth in total height (cm/y)	3.4	1.8, 4.9	3.5	2.2, 4.8	0.56	0.25, 0.87	1.69	-3.51, 6.88	-2.97	-5.75, -0.20
Growth in leg length (cm/y)	3.0	1.1, 4.8	4.0	2.4, 5.6	0.38	-0.04, 0.81	-1.12	-7.62, 5.38	-1.37	-4.89, 2.15
Growth in trunk length (cm/y)	5.2	3.3, 7.1	4.8	3.1, 6.4	0.86	0.50, 1,22	7.32	0.95,13.70	-4.31	-7.73, -0.90

Abbreviations: CI, confidence interval; HDL, high-density lipoprotein; HOMA-IR, homeostatic model assessment of insulin resistance. The Associations are adjusted for child's age at both the early and mid-childhood visit, race/ethnicity, baseline height component (early childhood), rate of growth in other height component, maternal height and pre-pregnancy body mass index, education and marital status in pregnancy, and paternal height and body mass index (Model 4). Growth is calculated as the difference in the respective variable between early and mid-childhood visit, divided by the time elapsed in years.

**Fig 1:**
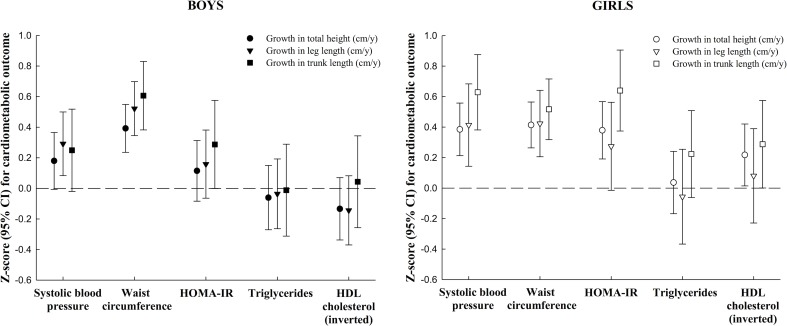
Associations of growth in total height, leg length and trunk length from early to mid-childhood with each of the five components of the mid-childhood cardiometabolic risk score in boys and girls (610 participants from Project Viva). Growth is expressed as cm/year. All outcomes are in z-scores. Associations are adjusted for child's age at the early (median age 3.2 years) and mid-childhood (median age 7.7 years) visit, race/ethnicity, baseline height component (early childhood), rate of growth in other height component, maternal height and pre-pregnancy body mass index, paternal height and body mass index, mother's education and marital status in pregnancy.

**Fig 2:**
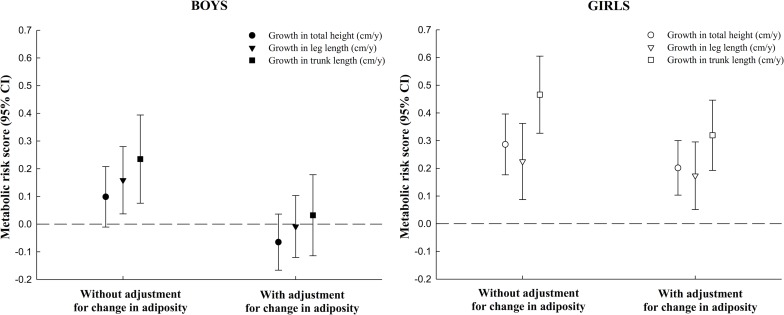
Influence of concurrent gain in adiposity on associations of growth in total height, leg length and trunk length from early to mid-childhood with mid-childhood cardiometabolic risk score in boys and girls (610 participants from Project Viva). Abbreviations: HOMA-IR, homeostatic model assessment of insulin resistance; HDL, high-density lipoprotein. Gain in adiposity is assessed as the change in sum of subscapular and triceps skinfold thickness (mm/y) and growth is expressed as cm/year. The cardiometabolic risk score is composed of the mean of five sex-specific internal z-scores for systolic blood pressure, waist circumference, log-transformed HOMA-IR, log-transformed triglycerides and inverted HDL-cholesterol. Associations are adjusted for child's age at the early (median age 3.2 years) and mid-childhood (median age 7.7 years) visit, race/ethnicity, baseline height component (early childhood), rate of growth in other height component, maternal height and pre-pregnancy body mass index, paternal height and body mass index, mother's education and marital status in pregnancy

In boys, it was especially the positive associations of growth in total height and its components with waist circumference along with the slightly weaker associations with systolic blood pressure and HOMA-IR that contributed to the higher cardiometabolic risk score in mid-childhood, whereas for girls the positive associations of growth in total height and its components with systolic blood pressure, waist circumference and HOMA-IR, almost equally contributed to the higher cardiometabolic risk score (Fig 1). The pattern was almost similar for boys and girls, but appeared stronger in girls. In girls, adjusting for change in adiposity attenuated the associations of total height and its components with cardiometabolic risk score with approximately 25%, whereas among boys all associations disappeared when adjusting for change in overall adiposity (Fig 2). The only associations that were not changed were associations of linear growth with systolic blood pressure among girls (results not shown).

## Discussion

In this longitudinal study of US children, faster linear growth as assessed by annual increases in total height, leg length, and trunk length from early to mid-childhood was associated with a higher cardiometabolic risk in mid-childhood, with strongest associations observed for increases in trunk length. These associations were primarily due to positive associations with three of the five cardiometabolic risk score components: systolic blood pressure, waist circumference, and HOMA-IR. The associations were considerably stronger in girls, who also had a higher mean level of HOMA-IR, than in boys. The associations were largely unchanged when accounting for parental anthropometrics, suggesting that the observed associations are primarily due to factors other than genetics. In boys, the associations were explained by relationships of linear growth with overall adiposity, whereas this was only partly the case in girls.

Prior work in Project Viva demonstrated that growth in trunk length from early to mid-childhood is associated with an increase in systolic blood pressure during the period [17]. In this study we extended those results by 1.) showing that linear growth in both sexes from early to mid-childhood is associated with a generally higher cardiometabolic risk, and 2.) trunk length growth is associated with an adverse cardiometabolic state more strongly than is increase in leg length.

In our prior work, we hypothesized that children with greater trunk length have higher blood pressures because an additional pressure is needed to overcome gravity to perfuse the brain [17]. Even though we still find the strongest associations with trunk length and cardiometabolic risk in the current study, we do not think that a higher hydrostatic pressure explains the associations with components other than systolic blood pressure. In addition, our finding of change in overall adiposity explaining the associations with systolic blood pressure in boys does not favor this hypothesis, rather it suggests that, at least in part, factors that promote linear growth also promote fat accumulation, which in turn lead to adverse cardiometabolic outcomes. The most important external factor influencing linear growth is nutrition, with protein being the most essential single nutrient. Protein intake is associated with different specific growth hormones, e.g. insulin-like growth factor 1, which in turn has been found to be associated with risk of cardiometabolic disease [25, 26]. It was however beyond the scope of our study to investigate potential mediation through protein intake or hormone levels in childhood.

In contrast to our findings, among pre-pubertal children in 1937–1939 in the Boyd Orr cohort, leg length, but not trunk length, was associated with CHD in adulthood [15, 16]. Those results, however, were not based on repeated measurements or adjusted for adiposity. It is possible that underlying factors for the association between leg length and CHD seen in the historical Boyd Orr cohort, e.g. malnourishment, were not present to the same extent in our modern, well-nourished cohort. Moreover, it is possible that surrogate outcomes of cardiometabolic risk in childhood are not perfect predictors of adult cardiovascular disease even though a compound cardiometabolic risk score has been found to be a valid tool for detecting subclinical atherosclerosis in children [27] as well as predicting type 2 diabetes and cardiovascular outcomes in adults [22, 28].

Some studies among adults have found that trunk length is associated with insulin resistance and type 2 diabetes [8, 29, 30], a condition that is increasing in children and adolescents, especially among girls [31]. In one study the association persisted after adjusting for current BMI [29], but in another study the association disappeared when adjusting for waist circumference [30]. In our study, growth in trunk length was associated with higher HOMA-IR, and this association was explained by concurrent adiposity in boys, but only partially in girls. This finding aligns with findings in the Early Bird study, a cohort study of 300 schoolchildren in the UK, where higher levels of HOMA-IR existed among taller and faster- growing girls [23]. As in our study, girls’ HOMA-IR levels were higher than in boys at age 5 years, regardless of levels of adiposity and physical activity [24]. The authors suggested that HOMA-IR is a marker of linear growth as well as of adiposity, and that pre-pubertal girls are at higher risk of developing type 2 diabetes early in life because they are intrinsically more insulin resistant than boys [23, 24].

Other authors hypothesized that early onset of puberty explains why both faster pre-pubertal linear growth and shorter leg length in adulthood are associated with later cardiometabolic diseases [6]. Girls reach puberty earlier than boys, which might explain the higher insulin resistance among girls. Our results do not support that hypothesis, as adjusting for puberty status did not change the results. However, only 6.9% boys and 11.0% girls had signs of having entered puberty, so longer follow-up is needed to further address this issue. Furthermore, adipose pre-pubertal children also experience faster linear growth, but it is still unclear whether their final height will be greater than that of their non-adipose peers [32–34].

Strengths of this study include repeated measurements of detailed anthropometry, consideration of parental anthropometry, and the availability of several different childhood cardiometabolic biomarkers. Although prospective evidence suggests that height components are related to later cardiometabolic disease, most research that includes markers of cardiometabolic health has been cross-sectional and has examined individual markers rather than a summary cardiometabolic risk score. Furthermore, we have not found any modern cohort studies investigating associations of childhood height components, or growth in height components, with cardiometabolic health.

There are also limitations to this study. Project Viva represents a relatively well-educated and high-income population despite its diverse racial and ethnic backgrounds, which means that findings may not be generalizable to lower socio-economic populations and settings in developing countries. Furthermore, we assumed, for practical reasons, that total height and sitting height provide sufficient information to partition stature into trunk and leg length. Calculating leg length from total height and sitting height, instead of measuring leg length from the greater trochanter to the floor, increases the measurement error as it is based on two measurements instead of one, which could bias the results for leg length towards the null. Moreover, buttock fatness may lead to overestimation of trunk length and consequently underestimation of leg length in overweight children [35] which could have biased our results in favor of stronger associations for trunk versus leg length. By adjusting for overall adiposity gain, however, we minimize this issue. We assumed the same rate of linear growth for every year between the two visits. This assumption is reasonable in a cohort of largely pre-pubertal children [36].

## Conclusions

More rapid linear growth from early to mid-childhood, particularly in trunk length, was associated with higher cardiometabolic risk in mid-childhood, indicating that it may be of great importance to also consider linear growth when evaluating cardiometabolic biomarkers in childhood. The higher cardiometabolic risk was due to associations with not only systolic blood pressure but also waist circumference and HOMA-IR, suggesting a potential higher risk of developing hypertension, type II diabetes, and possibly cardiovascular disease later in life. The associations were explained by relationships of linear growth with overall adiposity in boys, but this was only partly the case for girls.

## Supporting Information

S1 Table Multivariable linear regression models showing associations of growth in total height and its components from early to mid-childhood with cardiometabolic risk score in mid-childhood without adjusting for baseline total height or height component (610 participants from Project Viva). Abbreviations: CI, confidence interval. Model 1: Adjusted for child’s age at the early and mid-childhood visit and child’s race/ethnicity. Model 2: Model 1 + rate of growth in other height component (i.e. rate of growth in leg length adjusted for rate of growth in trunk length and vice versa). Model 3: Model 2 + maternal pre-pregnancy body mass index and height and paternal body mass index and height. Model 4: Model 3 + maternal education and marital status in pregnancy. Growth is calculated as the difference in the respective variable between early and mid-childhood visit, divided by the time elapsed in years. The cardiometabolic risk score is composed of the mean of five sex-specific internal z-scores for systolic blood pressure, waist circumference, log-transformed HOMA-IR, log-transformed triglycerides and inverted HDL-cholesterol.
(DOCX)

S2 Table Associations of growth in total height and its components from early to mid-childhood with change in adiposity (610 participants from Project Viva). Abbreviations: CI, confidence interval; y, year. All estimates are adjusted for child's age at both the early and mid-childhood visit, race/ethnicity, baseline height component (early childhood), rate of growth in other height component, maternal height and pre-pregnancy body mass index, education and marital status in pregnancy, and paternal height and body mass index (Model 4). Growth/Change is calculated as the difference in the respective variable between early and mid-childhood visit, divided by the time elapsed in years.
(DOCX)

S3 Table Number of imputed values for parental and child characteristics of 610 Project Viva participants as well as characteristics of 470 participants with unimputed information on all variables. Abbreviations: BMI, body mass index; HDL, high-density lipoprotein; HOMA-IR, homeostatic model assessment of insulin resistance; y, year. Change is calculated as the difference in the respective variable between early and mid-childhood visit, divided by the time elapsed in years. The cardiometabolic risk score is composed of the mean of five sex-specific internal z-scores for systolic blood pressure, waist circumference, log-transformed HOMA-IR, log-transformed triglycerides and inverted HDL-cholesterol.
(DOCX)

S4 Table Additional descriptive characteristics of 610 Project Viva Participants. Abbreviations: n, number; SD, standard deviation; SE, standard error; y, year
(DOCX)

S5 Table Pearson correlation matrix of early and mid-childhood anthropometrics for 610 Project Viva participants. (DOCX)
