# Supplementary material for: Growth in Total Height and Its Components and Cardiometabolic Health in Childhood
Source: PLoS One. 2016 Sep 22;11(9):e0163564. doi: 10.1371/journal.pone.0163564 (PMC5033234; doi:10.1371/journal.pone.0163564)
Supplement: S1 Table — Abbreviations: CI, confidence interval. Model 1: Adjusted for child’s age at the early and mid-childhood visit and child’s race/ethnicity. Model 2: Model 1 + rate of growth in other height component (i.e. rate of growth in leg length adjusted for rate of growth in trunk length and vice versa). Model 3: Model 2 + maternal pre-pregnancy body mass index and height and paternal body mass index and height. Model 4: Model 3 + maternal education and marital status in pregnancy. Growth is calculated as the difference in the respective variable between early and mid-childhood visit, divided by the time elapsed in years. The cardiometabolic risk score is composed of the mean of five sex-specific internal z-scores for systolic blood pressure, waist circumference, log-transformed HOMA-IR, log-transformed triglycerides and inverted HDL-cholesterol. (DOCX) [file pone.0163564.s001.docx]

| **S1 Table.** M**ultivariable linear regression models showing associations of growth in total height and its components from early to mid-childhood with cardiometabolic risk score in mid-childhood without adjusting for baseline total height or height component (610 participants from Project Viva).** Abbreviations: CI, confidence interval. Model 1: Adjusted for child’s age at the early and mid-childhood visit and child’s race/ethnicity. Model 2: Model 1 + rate of growth in other height component (i.e. rate of growth in leg length adjusted for rate of growth in trunk length and vice versa). Model 3: Model 2 + maternal pre-pregnancy body mass index and height and paternal body mass index and height. Model 4: Model 3 + maternal education and marital status in pregnancy. Growth is calculated as the difference in the respective variable between early and mid-childhood visit, divided by the time elapsed in years. The cardiometabolic risk score is composed of the mean of five sex-specific internal z-scores for systolic blood pressure, waist circumference, log-transformed HOMA-IR, log-transformed triglycerides and inverted HDL-cholesterol. | | | | | | | | |
| --- | --- | --- | --- | --- | --- | --- | --- | --- |
|  | **Change in cardiometabolic risk score (95% CI) per 1 cm annual growth** | | | | | | | |
|  | Model 1 | | Model 2 | | Model 3 | | Model 4 | |
|  | β | 95 % CI | Β | 95 % CI | β | 95 % CI | β | 95 % CI |
| **Boys (n=315)** |  |  |  |  |  |  |  |  |
| Growth in total height (cm/y) | 0.20 | 0.10, 0.30 | -- | -- | 0.18 | 0.07, 0.28 | 0.18 | 0.08, 0.29 |
| Growth in leg length (cm/y) | 0.18 | 0.06, 0.30 | 0.17 | 0.05, 0.28 | 0.17 | 0.05, 0.29 | 0.17 | 0.05, 0.30 |
| Growth in trunk length (cm/y) | 0.17 | 0.00, 0.33 | 0.20 | 0.04, 0.36 | 0.20 | 0.04, 0.36 | 0.20 | 0.04, 0.36 |
| **Girls (n=295)** |  |  |  |  |  |  |  |  |
| Growth in total height (cm/y) | 0.35 | 0.25, 0.45 | -- | -- | 0.34 | 0.24, 0.44 | 0.34 | 0.23, 0.44 |
| Growth in leg length (cm/y) | 0.23 | 0.10, 0.37 | 0.27 | 0.14, 0.40 | 0.25 | 0.12, 0.38 | 0.25 | 0.11, 0.39 |
| Growth in trunk length (cm/y) | 0.43 | 0.29, 0.58 | 0.45 | 0.31, 0.59 | 0.44 | 0.30, 0.58 | 0.44 | 0.30, 0.58 |
