# Supplementary material for: Growth in Total Height and Its Components and Cardiometabolic Health in Childhood
Source: PLoS One. 2016 Sep 22;11(9):e0163564. doi: 10.1371/journal.pone.0163564 (PMC5033234; doi:10.1371/journal.pone.0163564)
Supplement: S2 Table — Abbreviations: CI, confidence interval; y, year. All estimates are adjusted for child's age at both the early and mid-childhood visit, race/ethnicity, baseline height component (early childhood), rate of growth in other height component, maternal height and pre-pregnancy body mass index, education and marital status in pregnancy, and paternal height and body mass index (Model 4). Growth/Change is calculated as the difference in the respective variable between early and mid-childhood visit, divided by the time elapsed in years. (DOCX) [file pone.0163564.s002.docx]

| **S2 Table.** **Associations of growth in total height and its components from early to mid-childhood with change in adiposity (610 participants from Project Viva).** Abbreviations: CI, confidence interval; y, year. All estimates are adjusted for child's age at both the early and mid-childhood visit, race/ethnicity, baseline height component (early childhood), rate of growth in other height component, maternal height and pre-pregnancy body mass index, education and marital status in pregnancy, and paternal height and body mass index (Model 4). Growth/Change is calculated as the difference in the respective variable between early and mid-childhood visit, divided by the time elapsed in years. | | | | |
| --- | --- | --- | --- | --- |
|  | Change in supscapular+triceps skinfold thickness (mm/y) per 1 cm annual growth | | Difference in supscapular+triceps skinfold thickness (mm) per 1 cm annual growth | |
|  | β | 95% CI | β | 95% CI |
| **Boys (n=315)** |  | |  | |
| Growth in total height (cm/y) | 0.81 | 0.54, 1.08 | 3.36 | 2.14, 4.59 |
| Growth in leg length (cm/y) | 0.81 | 0.52, 1.10 | 3.26 | 1.93, 4.60 |
| Growth in trunk length (cm/y) | 1.00 | 0.62, 1.38 | 4.59 | 2.84, 6.34 |
| **Girls (n=295)** |  |  |  |  |
| Growth in total height (cm/y) | 0.50 | 0.19, 0.81 | 2.58 | 1.12, 4.04 |
| Growth in leg length (cm/y) | 0.30 | -0.08, 0.69 | 1.66 | -0.15, 3.47 |
| Growth in trunk length (cm/y) | 0.89 | 0.48, 1.29 | 4.19 | 2.31, 6.08 |
