# Supplementary material for: Growth in Total Height and Its Components and Cardiometabolic Health in Childhood
Source: PLoS One. 2016 Sep 22;11(9):e0163564. doi: 10.1371/journal.pone.0163564 (PMC5033234; doi:10.1371/journal.pone.0163564)
Supplement: S3 Table — Abbreviations: BMI, body mass index; HDL, high-density lipoprotein; HOMA-IR, homeostatic model assessment of insulin resistance; y, year. Change is calculated as the difference in the respective variable between early and mid-childhood visit, divided by the time elapsed in years. The cardiometabolic risk score is composed of the mean of five sex-specific internal z-scores for systolic blood pressure, waist circumference, log-transformed HOMA-IR, log-transformed triglycerides and inverted HDL-cholesterol. (DOCX) [file pone.0163564.s003.docx]

| **S3 Table.** **Number of imputed values for parental and child characteristics of 610 Project Viva participants as well as characteristics of 470 participants with unimputed information on all variables.** Abbreviations: BMI, body mass index; HDL, high-density lipoprotein; HOMA-IR, homeostatic model assessment of insulin resistance; y, year. Change is calculated as the difference in the respective variable between early and mid-childhood visit, divided by the time elapsed in years. The cardiometabolic risk score is composed of the mean of five sex-specific internal z-scores for systolic blood pressure, waist circumference, log-transformed HOMA-IR, log-transformed triglycerides and inverted HDL-cholesterol. | | | | |
| --- | --- | --- | --- | --- |
|  |  |  | **470 paticipants with unimputed data** | |
|  | **n (non-missing)** | **n (imputed)** | **Boys (n=242)** | **Girls (n=228)** |
|  |  |  | **% or Mean (SD)** | |
| **Parental and family characteristics** |  |  |  |  |
| Maternal age at enrollment, years | 610 | 0 | 32.1 (5.4) | 32.5 (5.2) |
| Maternal height, cm | 610 | 0 | 165.8 **(**7.3) | 165.1 **(**6.8) |
| Maternal pre-pregnancy BMI, kg/m^2^ | 610 | 0 | 24.7 **(**5.3) | 24.8 **(**5.0) |
| Paternal height, cm | 600 | 10 | 179.1 (7.7) | 179.2 **(**7.8) |
| Paternal BMI, kg/m^2^ | 586 | 24 | 25.9 **(**3.6) | 27.0 **(**3.8) |
| Maternal education | 610 | 0 |  |  |
| < College grad |  |  | 35.5% | 27.6% |
| ≥ College grad |  |  | 64.5% | 72.4% |
| Marital status | 609 | 1 |  |  |
| Single |  |  | 9.1% | 10.6% |
| Married/cohabiting |  |  | 90.9% | 89.4% |
| Annual household income | 572 | 38 |  |  |
| < $70,000 |  |  | 38.8% | 36.1% |
| > $70,000 |  |  | 61.2% | 63.9% |
| **Early childhood visit** (median age 3.2 years) |  |  |  |  |
| Child’s race/ethnicity | 610 | 0 |  |  |
| Black |  |  | 19.8% | 15.8% |
| Hispanic |  |  | 5.0% | 4.0% |
| Asian |  |  | 62.0% | 66.2% |
| White |  |  | 2.9% | 2.6% |
| Other |  |  | 10.3% | 11.4% |
| Total height, cm | 602 | 8 | 98.1 (4.5) | 96.9 (4.7) |
| Leg length, cm | 600 | 10 | 42.2 (2.8) | 41.7 (2.9) |
| Trunk length, cm | 600 | 10 | 55.8 (2.6) | 55.2 (2.4) |
| Subscapular + triceps skinfold thicknesses, mm | 583 | 27 | 15.9 (3.9) | 17.4 (4.6) |
| **Mid-childhood visit** (median age 7.7 years) |  |  |  |  |
| Total height, cm | 610 | 0 | 128.9 (7.2) | 127.8 (7.1) |
| Leg length, cm | 609 | 1 | 60.6 (4.6) | 59.6 (4.5) |
| Trunk length, cm | 609 | 1 | 68.3 (3.4) | 68.1 (3.5) |
| Subscapular + triceps skinfold thickness, mm | 609 | 1 | 17.8 (8.3) | 22.0 (10.3) |
| Time between early and mid-childhood visit, years | 609 | 1 | 4.6 (0.8) | 4.5 (0.7) |
| Change in total height, cm/y | 602 | 8 | 6.7 **(**0.7) | 6.8 **(**0.7) |
| Change in leg length, cm/y | 599 | 11 | 4.0 **(**0.6) | 4.0 **(**0.5) |
| Change in trunk length, cm/y | 599 | 11 | 2.7 **(**0.4) | 2.8 **(**0.5) |
| Change in subscapular + triceps skinfold thickness^a^, mm/y | 583 | 27 | 0.3 **(**1.5) | 1.0 **(**1.9) |
| Pubertal status in mid-childhood, | 588 | 22 |  |  |
| No |  |  | 82.3% | 69.0% |
| Maybe (barely started) |  |  | 11.2% | 21.1% |
| Yes |  |  | 6.5% | 9.9% |
| Cardiometabolic outcomes |  |  |  |  |
| Systolic blood pressure, mmHg | 608 | 2 | 94.2 (8.6) | 94.2 (9.0) |
| Waist circumference, cm | 609 | 1 | 59.3 (8.1) | 60.0 (8.4) |
| HOMA-IR, units | 490 | 120 | 1.7 (1.5) | 2.0 (1.5) |
| Triglycerides, mg/dL | 548 | 62 | 57.7 (24.9) | 60.2 (25.0) |
| HDL-cholesterol, mg/dL | 548 | 62 | 58.8 (13.0) | 55.6 (13.9) |
| Cardiometabolic risk score | 477 | 133 | -0.03 (0.58) | -0.03 (0.64) |
