# Supplementary material for: Growth in Total Height and Its Components and Cardiometabolic Health in Childhood
Source: PLoS One. 2016 Sep 22;11(9):e0163564. doi: 10.1371/journal.pone.0163564 (PMC5033234; doi:10.1371/journal.pone.0163564)
Supplement: S4 Table — Abbreviations: n, number; SD, standard deviation; SE, standard error; y, year (DOCX) [file pone.0163564.s004.docx]

| **S4 Table. Additional descriptive characteristics of 610 Project Viva Participants.** Abbreviations: n, number; SD, standard deviation; SE, standard error; y, year | | | | | | | | | | | | |
| --- | --- | --- | --- | --- | --- | --- | --- | --- | --- | --- | --- | --- |
|  | **Boys (n=315)** | | | | | | **Girls (n=295)** | | | | | |
|  | **Mean** | **SD** | **SE** | **Min** | **Median** | **Max** | **Mean** | **SD** | **SE** | **Min** | **Median** | **Max** |
| **Early childhood visit** |  |  |  |  |  |  |  |  |  |  |  |  |
| Age, y | 3.3 | 0.4 | 0.02 | 2.9 | 3.2 | 5.9 | 3.3 | 0.3 | 0.02 | 3.0 | 3.1 | 6.2 |
| Total height, cm | 97.9 | 4.5 | 0.25 | 85.4 | 97.6 | 114.4 | 97.1 | 4.7 | 0.27 | 86.5 | 96.6 | 129.1 |
| Leg length, cm | 42.1 | 2.8 | 0.16 | 35.4 | 42.0 | 53.1 | 41.9 | 2.9 | 0.17 | 34.1 | 41.5 | 61.8 |
| Trunk length, cm | 55.8 | 2.6 | 0.15 | 48.2 | 55.8 | 62.9 | 55.3 | 2.5 | 0.15 | 48.9 | 55.4 | 67.3 |
| **Mid-childhood visit** |  |  |  |  |  |  |  |  |  |  |  |  |
| Age, y | 7.9 | 0.8 | 0.04 | 6.7 | 7.7 | 10.6 | 7.8 | 0.7 | 0.04 | 6.7 | 7.6 | 10.9 |
| Total height, cm | 129.1 | 7.2 | 0.41 | 109.3 | 128.5 | 163.1 | 128.3 | 7.9 | 0.46 | 110.9 | 127.7 | 159.7 |
| Leg length, cm | 60.6 | 4.7 | 0.26 | 49.1 | 59.9 | 85.4 | 60.1 | 5.1 | 0.30 | 49.4 | 59.7 | 86.6 |
| Trunk length, cm | 68.5 | 3.4 | 0.19 | 57.5 | 68.5 | 80.2 | 68.2 | 3.6 | 0.21 | 59.0 | 67.8 | 79.9 |
| **Between the two visits** |  |  |  |  |  |  |  |  |  |  |  |  |
| ∆time, y | 4.6 | 0.8 | 0.05 | 2.0 | 4.5 | 7.5 | 4.5 | 0.7 | 0.04 | 1.3 | 4.4 | 7.4 |
| ∆total height, cm/y | 6.7 | 0.7 | 0.04 | 2.2 | 6.8 | 8.7 | 6.9 | 0.7 | 0.04 | 5.1 | 6.8 | 9.5 |
| ∆leg length, cm/y | 4.0 | 0.6 | 0.03 | 0.2 | 4.0 | 5.8 | 4.0 | 0.5 | 0.03 | 2.1 | 4.0 | 6.7 |
| ∆trunk length, cm/y | 2.7 | 0.4 | 0.02 | 1.0 | 2.8 | 4.3 | 2.9 | 0.5 | 0.03 | 0.5 | 2.8 | 5.2 |
