# Supplementary material for: Growth in Total Height and Its Components and Cardiometabolic Health in Childhood
Source: PLoS One. 2016 Sep 22;11(9):e0163564. doi: 10.1371/journal.pone.0163564 (PMC5033234; doi:10.1371/journal.pone.0163564)
Supplement: S5 Table — (DOCX) [file pone.0163564.s005.docx]

| **S5 Table. Pearson correlation matrix of early and mid-childhood anthropometrics for 610 Project Viva participants.** | | | | | | | | | |
| --- | --- | --- | --- | --- | --- | --- | --- | --- | --- |
|  | **Spearman r** | | | | | | | | |
| **Anthropometric variables** | Leg length in early childhood | Trunk length in early childhood | Total height in early childhood | Leg length in mid-childhood | Trunk length in mid-childhood | Total height in mid-childhood | ∆leg length | ∆trunk length | ∆total height |
| Leg length in early childhood | 1.00 | 0.42 | 0.86 | 0.57 | 0.28 | 0.50 | 0.14 | 0.07 | 0.16 |
| Trunk length in early childhood | 0.42 | 1.00 | 0.82 | 0.43 | 0.60 | 0.55 | 0.46 | -0.17 | 0.25 |
| Total length in early childhood | 0.86 | 0.82 | 1.00 | 0.60 | 0.52 | 0.62 | 0.35 | -0.05 | 0.24 |
| Leg length in mid-childhood | 0.57 | 0.43 | 0.60 | 1.00 | 0.62 | 0.93 | 0.58 | -0.05 | 0.44 |
| Trunk length in mid-childhood | 0.28 | 0.60 | 0.52 | 0.62 | 1.00 | 0.86 | 0.28 | 0.40 | 0.49 |
| Total height in mid-childhood | 0.50 | 0.55 | 0.62 | 0.93 | 0.86 | 1.00 | 0.51 | 0.15 | 0.51 |
| ∆leg length | 0.14 | 0.46 | 0.35 | 0.58 | 0.28 | 0.51 | 1.00 | -0.07 | 0.76 |
| ∆trunk length | 0.07 | -0.17 | -0.05 | -0.05 | 0.40 | 0.15 | -0.07 | 1.00 | 0.60 |
| ∆total height | 0.16 | 0.25 | 0.24 | 0.44 | 0.49 | 0.51 | 0.76 | 0.60 | 1.00 |
